# *APOE* genotype and sex affect microglial interactions with plaques in Alzheimer’s disease mice

**DOI:** 10.1186/s40478-019-0729-z

**Published:** 2019-05-21

**Authors:** T. L. Stephen, M. Cacciottolo, D. Balu, T. E. Morgan, M. J. LaDu, C. E. Finch, C. J. Pike

**Affiliations:** 1 Leonard Davis School of Gerontology, University of Southern California, 3715 McClintock Avenue, Los Angeles, CA 90089-0191 USA; 2 Department of Anatomy and Cell Biology, University of Illinois at Chicago, Chicago, IL 60612 USA

**Keywords:** Alzheimer’s disease, Amyloid, Apolipoprotein E, Microglia, Plaques, Sex differences, TREM2

## Abstract

Microglia affect Alzheimer’s disease (AD) pathogenesis in opposing manners, by protecting against amyloid accumulation in early phases of the disease and promoting neuropathology in advanced stages. Recent research has identified specific microglial interactions with amyloid plaques that exert important protective functions including attenuation of early pathology. It is unknown how these protective microglial interactions with plaques are affected by apolipoprotein E (*APOE*) genotype and sex, two well-established AD risk factors that modulate microglial function. We investigated this question using quantitative confocal microscopy to compare microglial interactions with amyloid plaques in male and female EFAD mice across *APOE3* and *APOE4* genotypes at 6 months of age. We observed that microglial coverage of plaques is highest in male *APOE3* mice with significant reductions in coverage observed with both *APOE4* genotype and female sex. Plaque compaction, a beneficial consequence of microglial interactions with plaques, showed a similar pattern in which *APOE4* genotype and female sex were associated with significantly lower values. Within the plaque environment, microglial expression of triggering receptor expressed on myeloid cells 2 (TREM2), a known regulator of microglial plaque coverage, was highest in male *APOE3* mice and reduced by *APOE4* genotype and female sex. These differences in plaque interactions were unrelated to the number of microglial processes in the plaque environment across groups. Interestingly, the pattern of amyloid burden across groups was opposite to that of microglial plaque coverage, with *APOE4* genotype and female sex showing the highest amyloid levels. These findings suggest a possible mechanism by which microglia may contribute to the increased AD risk associated with *APOE4* genotype and female sex.

**Electronic supplementary material:**

The online version of this article (10.1186/s40478-019-0729-z) contains supplementary material, which is available to authorized users.

## Introduction

The neuropathology of AD is characterized primarily by the region-specific accumulation of amyloid beta (Aβ) into senile plaques and hyperphosphorylated tau into neurofibrillary tangles. Both plaques and tangles are widely hypothesized to contribute to the neurodegenerative changes that occur in AD and manifest clinically as dementia [56]. AD neuropathology also involves several other significant components, including microglial activation, that are associated with disease progression. While it has long been known that activated microglia co-localize with Aβ plaques [20], as noted by Alzheimer himself [47], their roles remain incompletely defined. Microglia exhibit a broad range of actions implicated in both normal neural function [6, 13, 18] and the development of disease [5, 19]. In the context of AD, activated microglia have been theorized to exert dual effects on disease progression, promoting AD by driving neuroinflammation while also attenuating pathogenesis as a result of phagocytic actions [29]. Moreover, recent observations suggest that microglia interact with plaques to form a barrier that reduces the outward extension of Aβ fibrils, which may protect nearby neurites from damage [4, 9, 21, 25, 27, 52, 58, 61, 63, 64].

The regulation of microglial-plaque associations is a topic of high importance. A key molecule in the regulation of these interactions is triggering receptor expressed on myeloid cells 2 (TREM2), a microglial cell surface receptor of the immunoglobulin superfamily that senses damage in the central nervous system [42, 57]. TREM2 activation is essential for immune function in the brain, promoting proliferation, tropism and survival of microglia [38, 52]. Heterozygous TREM2 mutations, which yield partial loss of function, confer higher AD risk [14, 23, 24, 46] and are associated with reduced interactions of microglia with plaques [63]. Rodent studies confirm that microglial-plaque interactions are TREM2-dependent. Specifically, TREM2 deficiency and haploinsufficiency in mouse models of AD are associated with disrupted clustering of microglia around Aβ plaques [21, 53, 57, 58] and diminished ability of microglia to form barriers around amyloid deposits, compact plaques, and reduce plaque-associated dystrophic neurites [63].

Apolipoprotein E (*APOE*) genotype, the most widely shared genetic risk factor for late-onset AD [16, 28], is a strong candidate regulator of TREM2-dependent microglial interactions with amyloid plaques. First, *APOE* genotype is an important modulator of microglial activation, with the AD-associated *APOE* ε4 allele (*APOE4*) linked with increased microgliosis and neuroinflammation [50]. Second, apoE is a ligand for TREM2 [2, 3, 61] that directly and/or indirectly activates TREM2-mediated signaling pathways, including those that induce phagocytosis and anti-inflammatory cascades [22, 53]. Third, cell culture evidence suggests that *APOE4* is associated with greater depletion of TREM2 expression following acute immune challenge [31], suggesting perhaps that *APOE4* may diminish TREM2-mediated actions. Whether interactions between *APOE* and TREM2 extend to microglial plaque interactions is unknown.

Our current study investigates the effects of *APOE* genotype on TREM2-dependent microglial interactions with plaques. We utilized the EFAD transgenic mouse model of AD, which includes hemizygous expression of 5xFAD model with knock-in of homozygous human *APOE3* or *APOE4* [62]. Because sex significantly affects *APOE4* risk for AD in humans [1, 11, 37] and AD-related pathology in transgenic mice [7], and because sex regulates microglia phenotype [55], we also included sex as a modulating variable. Our results indicate that TREM2-dependent microglial interactions with plaques are significantly affected by both *APOE* genotype and sex with indices of microglial interactions showing the poorest outcomes with *APOE4* genotype and female sex. These findings identify a new role for *APOE* genotype in regulation of microglia and AD pathogenesis and highlight the importance of sex as a modulator of these relationships.

## Materials and methods

### Animals

EFAD mice (5xFAD^+/−^ *APOE*^+/+^) are hemizygous for 5xFAD, a transgenic mouse model of AD with 5 familial AD transgenes, and homozygous for knock-in of human *APOE3* (E3FAD) or *APOE4* (E4FAD) on a C57BL/6 J genetic background [62]. Four groups were studied: male E3FAD (*n* = 7), female E3FAD (*n* = 7), male E4FAD (*n* = 5), and female E4FAD (*n* = 6). Mice were euthanized at 6 months of age, a time point associated with relatively early but significant levels of AD-related neuropathology [49, 62]. Mice were perfused transcardially with phosphate-buffered saline (pH 7.4), their brains were harvested, then bisected in the sagittal plane and fixed in 4% paraformaldehyde for 48 h.

### Histochemistry

Brain sections (40 μm) were cut using a vibratome and stained using modifications of standard procedures previously described [33]. Staining was performed in a limited number of batches that were balanced across experimental groups. For immunohistochemistry, sections were subjected to heat-mediated antigen retrieval with 10 mM EDTA for 10 min at 95 °C. Endogenous peroxidases were blocked by 3% H_2_O_2_ and 10% methanol in Tris-buffered saline (TBS, 30 min at room temperature). Sections were permeabilized in 0.1% Triton X-100 for 15 min, blocked by a 30 min incubation in blocking buffer (TBS with 3% bovine serum albumin and 0.1% Triton X-100), followed by incubation at 4 °C with primary antibodies (diluted in blocking buffer) against the microglial marker ionized calcium binding adaptor molecule 1 (Iba1) (WAKO rabbit; 1:500 dilution) and/or TREM2 (R&D Systems; sheep 1:500 dilution) for 2–3 days. After washing, sections were incubated with Alexa fluorophore-conjugated secondary antibodies (Invitrogen; anti-rabbit and anti-sheep) diluted 1:500 in blocking buffer for 1–2 days. To label amyloidogenic plaques, immunostained sections were incubated with 0.5% Thioflavin S (ThioS; Sigma-Aldrich) for 10 min and subsequently washed sequentially with 70% ethanol, 50% ethanol, and purified H_2_0 before mounting on glass slides with VECTASHIELD® Antifade mounting media (Vector Labs).

### Microscopy and image analyses

Images were captured using a confocal microscope (Zeiss Laser Scanning Microscope-780 upright microscope) with Zeiss ZEN imaging software by a researcher blinded to experimental groups. Laser and detector settings were unchanged across acquisition sessions. Images of sections labeled with ThioS, anti-Iba1 and/or anti-TREM2 were collected in z-stacks at 3 μm intervals; for high-resolution images optimal stack section depths of 0.4 μm were used. A 63x oil immersion objective (1.4 NA) was used to acquire region of interest (ROI) stacks (192.8 μm × 192.8 μm, 512 × 512 pixels, 16 bit). Because levels of amyloid pathology adequate for quantification across all groups were observed only in the subiculum (Additional file 1: Figure S1), imaging analyses were restricted to hippocampal regions of subiculum and the adjacent cornu ammonis 1–3 (referred to as hippocampus, HPC). Non-overlapping ROIs (≥ 3 per section) were taken to sample the majority of pathology as well as adjacent HPC areas lacking pathology. The proportion of ROIs utilized for plaque analyses that were captured in the subiculum were similar across groups: 83% for E3FAD males, 83% for E3FAD females, 87% for E4FAD males, and 91% for E4FAD females (remaining proportion of ROIs were from cornu ammonis 1–3). Image analyses were performed in a user-blinded manner with a custom ImageJ plugin [48] and de-noised using background subtraction rolling ball radius of 50 pixels. Maximum projections were used for analysis (unless otherwise stated). For microglial interactions with plaques, all plaques in collected images were analyzed if they satisfied the following criteria: > 4 μm in diameter, not overlapping with other plaques and captured entirely within the ROI. Consistent with described methodology [9], microglial plaque coverage was quantified by manually identifying the intersections between the tips of microglia processes and the plaque perimeter. The proportion of the plaque perimeter covered by microglia processes was calculated by summing the arcs of plaque perimeter across 3-dimensional stacks in close contact (within 2 μm) with Iba1-immunolabeled cells (~ 20 plaques/group). Only microglial processes were counted in the plaque coverage analysis and any overlap with microglial cell bodies was not included. Plaque perimeters were manually determined by outlining each plaque in ImageJ. Plaque circularity, a measure of plaque compaction, was calculated as previously described [63] using the formula circularity = 4π x area / (perimeter)^2^. For microglia process ramifications, relative to plaque distance, Sholl analysis was performed using ImageJ. The number of Iba1-immunreactive process intersections was calculated at 10 μm intervals from the center of singular plaque regions (somas were omitted manually from these analyses). For immunohistochemical load quantification (ThioS, Iba1, and TREM2), images were converted to 8-bit grayscale using ImageJ, thresholded and normalized to the total ROI area (% of total area). Data represents averages of all ROIs for each animal (total HPC including subiculum). To determine microglial-specific TREM2 levels, the TREM2 signal was measured, as described above, and subtracted from background levels (i.e., removing Iba1 negative soluble TREM2) within the same ROI. Separate high-resolution z-stacks, with an optimal step size, were added to determine the levels of TREM2 in Iba1-positive microglia proximal to regions of plaque contact normalized to regions where there was no plaque contact (distal; within 30 μm of a plaque). The proximal/distal TREM2 ratio was calculated by dividing the TREM2 signal (co-localized with Iba1 staining) proximal to plaque-contact sites (within 5 μm) by the TREM2 signal (co-localized with Iba1 staining) distal to plaque-contact sites. Thus, a higher ratio reflects elevated TREM2 in Iba1 processes in close association with plaque staining. Microglial soma size was measured by manually identifying, outlining, and measuring Iba1-immunoreactive cell bodies (~ 60 cells/group) using ImageJ.

### Statistics

Data were statistically assessed using two-way analysis of variance (ANOVA), with *APOE* genotype and sex as independent variables, followed by Tukey post-hoc test for pairwise multiple comparisons, unless otherwise stated. Linear regression was used to analyze relationships between plaque perimeter and microglial plaque coverage, as well as microglial process number as a function of distance from plaques (Sholl analysis). Statistical analyses were performed using Prism version 8.0.1 (GraphPad Software, Inc.). All data are presented as mean ± SEM. For all statistical tests, *p* values less than 0.05 were considered significant.

## Results

### *APOE* genotype and sex are associated with microglial interactions with amyloid plaques

**Fig. 1:**
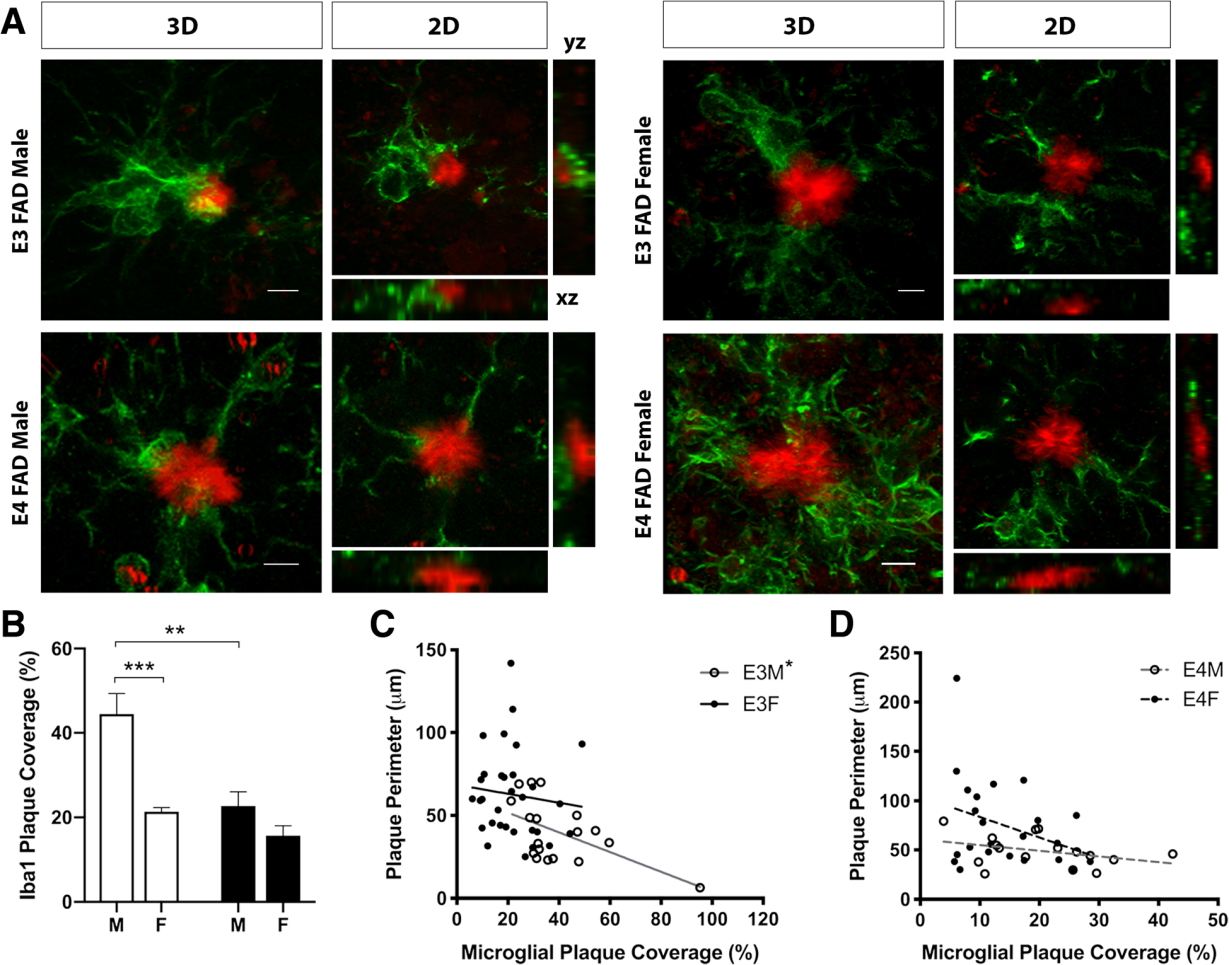
Microglial plaque coverage in EFAD mice is diminished by *APOE4* and female sex. **a** Representative images show ThioS-positive plaques (red) and Iba1-immunoreactive microglia (green) in the HPC of EFAD mice at 6 months of age. 3D images show composite, stacked images across the z-axis, whereas 2D images show individual z-sections with corresponding labeling across x- and y-axes. Scale bars = 5 μm. **b** Quantification of Iba1-immunoreactive processes in contact with ThioS-positive amyloid deposits (% plaque coverage) in male (M) and female (F) EFAD mice with *APOE3* (open bars) and *APOE4* (filled bars) genotypes. **c-d** Correlation plots of plaque perimeter in relation to microglial plaque coverage in *APOE3* (**c**) and *APOE4* (**d**) male and female mice. Numbers of mice analyzed per group are as follows: male E3FAD (*n* = 6), female E3FAD (*n* = 5), male E4FAD (*n* = 5), and female E4FAD (*n* = 6). * denotes *p* < 0.05, ** denotes *p* < 0.01 *** denotes *p* < 0.001

To explore the effects of *APOE* genotype and sex on interactions of microglia with amyloid plaques, high-resolution confocal images were used to capture microglia associated with ThioS-labeled deposits in EFAD mice (Fig. 1a). The percentage of individual plaque perimeters in close proximity with Iba1-immunolabeled microglial processes, termed plaque coverage, was quantified across z-stacks (Fig. 1b). There was a significant main effect of *APOE* genotype (Fig. 1b, *p* = 0.0007), with higher plaque coverage in E3FAD males than in E4FAD males. Further, there was a significant main effect of sex (Fig. 1b, *p* = 0.0004), where microglial coverage of plaques was two-fold greater in male E3FAD than in female E3FAD mice. In addition, there was a significant interaction between genotype and sex (Fig. 1b, *p* = 0.03) such that the sex difference in plaque coverage was apparent only in E3FAD mice. The level of plaque coverage by microglial processes varied inversely with plaque perimeter for male E3FAD (Fig. 1c, *p* = 0.02), but not for female E3FAD mice (Fig. 1c) or male and female E4FAD mice (Fig. 1d). Specifically, in male E3FAD mice, increasing levels of microglial process interactions with plaques were associated with reduced plaque size. This observation is consistent with prior findings that increased plaque coverage by microglial processes contributes to plaque compaction [63].

### *APOE* genotype and sex affect plaque compaction

**Fig. 2:**
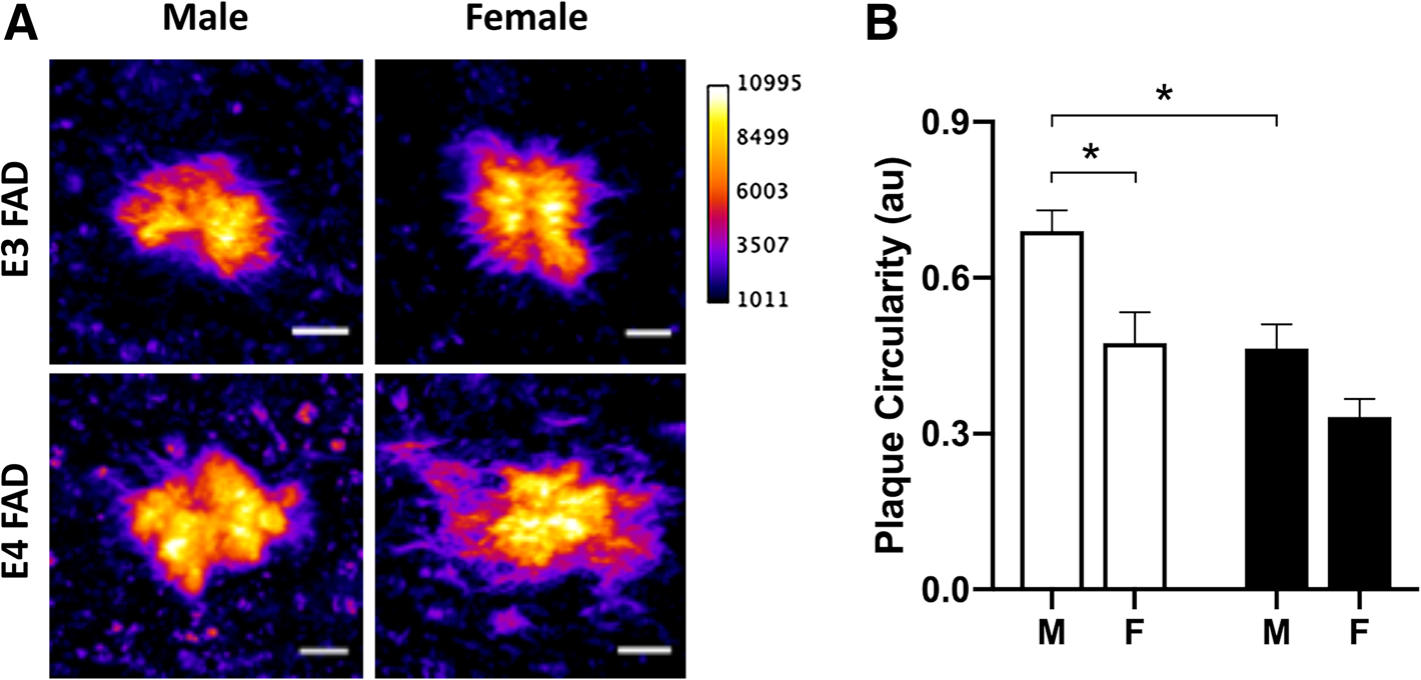
Effects of *APOE* genotype and sex on amyloid plaque dynamics. **a** Representative pseudo-colored images show relative circularity of ThioS-positive plaques, with intensity of labeling indicated by brightness (purple/black = low and yellow/white = high), across sex and *APOE* genotype. Scale bars = 5 μm. **b** Quantification of plaque circularity in male (M) and female (F) EFAD mice with *APOE3* (open bars) and *APOE4* (filled bars) genotypes. Numbers of mice analyzed per group are as follows: male E3FAD (*n* = 6), female E3FAD (*n* = 5), male E4FAD (*n* = 5), and female E4FAD (*n* = 6). * denotes *p* < 0.05

We subsequently investigated how differences in plaque coverage by microglia might impact the morphology of amyloid plaques. Quantitative confocal analysis of plaque compaction was calculated using a formula that measures the spherical nature of individual plaques while controlling for potential differences in plaque size that are expected across groups with different levels of pathology (Fig. 2a). In parallel to our findings on microglial coverage of plaques, we observed significant main effects of both *APOE* genotype and sex (Fig. 2b *p* = 0.0006 and *p* = 0.001, respectively) on plaque circularity, where the highest levels of plaque compaction were found in male E3FAD mice.

### *APOE* genotype and sex are associated with microglial TREM2 expression near amyloid plaques

**Fig. 3:**
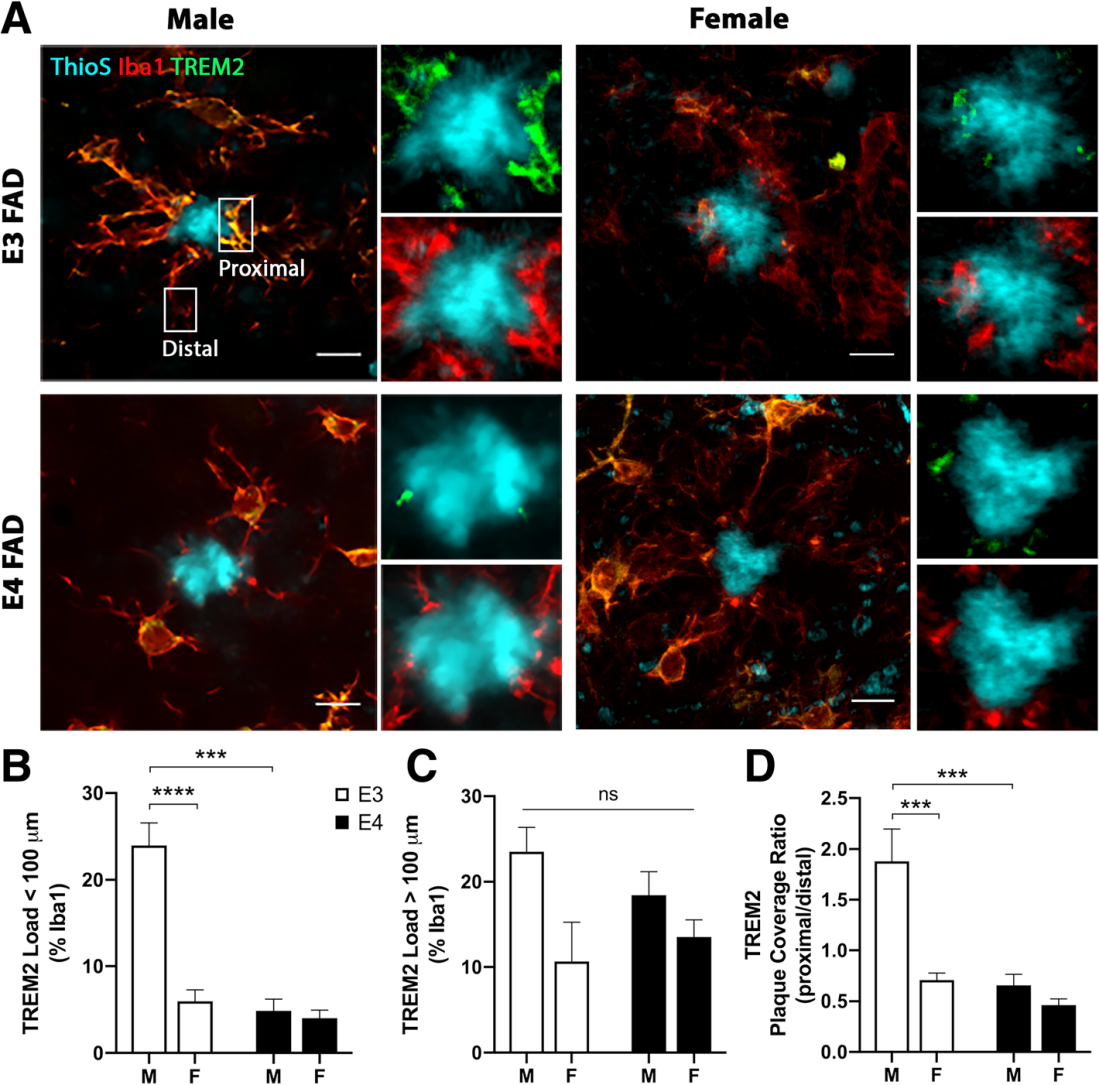
Effects of *APOE4* and sex on TREM2 expression levels near plaques. **a** Representative images showing ThioS-positive plaques (cyan) and Iba1-immunoreactive microglia (red) merged with TREM2 (green; with orange reflecting high co-localization with Iba1) in the HPC of EFAD mice. Scale bars = 10 μm. **b** Quantification of TREM2 load in the HPC in male (M) and female (F) EFAD mice with *APOE3* (open bars) and *APOE4* (filled bars) genotypes. **c** TREM2 load in plaque negative regions (> 100 μm away). **d** TREM2 intensity in Iba1-immunoreactive microglia proximal to sites of microglia-plaque interaction (normalized to distal regions). Numbers of mice analyzed per group are as follows: (**b**-**c**) male E3FAD (*n* = 6), female E3FAD (*n* = 7), male E4FAD (*n* = 5), and female E4FAD (*n* = 6). **d** male E3FAD (*n* = 4), female E3FAD (*n* = 6), male E4FAD (*n* = 4), and female E4FAD (*n* = 5). *** denotes *p* < 0.001, **** denotes *p* < 0.0001

TREM2-mediated signaling is required for microglial interactions with plaques. For example, a recent study of microglia in close proximity to amyloid deposits showed that TREM2 labeling is increased in processes that interact with plaques but not in processes that fail to interact [63]. We examined whether *APOE* genotype and/or sex affect the levels and cellular localization of TREM2 within microglia using HPC sections immunolabeled for TREM2 and Iba1 and counterstained with ThioS (Fig. 3a). Overall, TREM2 expression levels appeared to be highest in male E3FAD mice (Fig. 3a). Quantification of TREM2 labeling (co-localized with Iba1 immunoreactivity) within plaque regions (< 100 μm) revealed significant main effects of *APOE* genotype and sex (Fig. 3b, *p* < 0.0001 and *p* < 0.0001, respectively), showing that TREM2 levels were two-fold higher in E3FAD males than in E3FAD females and higher relative to E4FAD mice of both sexes (Fig. 3b). Further, there was an interaction between genotype and sex (Fig. 3b, *p* = 0.0002) such that the sex difference in TREM2 load was apparent only in E3FAD mice. These relationships depended on the plaque environment since there was no significant difference across groups in TREM2 levels in areas > 100 μm from plaque regions (Fig. 3c). Because TREM2 labeling is highest at sites of activation [63], we also compared the ratio of TREM2 labeling in processes proximal versus distal to plaques within individual Iba1-labeled cells. A similar pattern was observed in which proximal/distal levels of TREM2 labeling revealed significant main effects of *APOE* genotype and sex (Fig. 3d, *p* = 0.0002 and *p* = 0.0004, respectively) as well as a significant group interaction (Fig. 3d, *p* = 0.005), with levels again being highest in male E3FAD mice and no significant differences among the other three groups (Fig. 3d).

### *APOE* genotype and sex are not associated with microglial process number near amyloid plaques

**Fig. 4:**
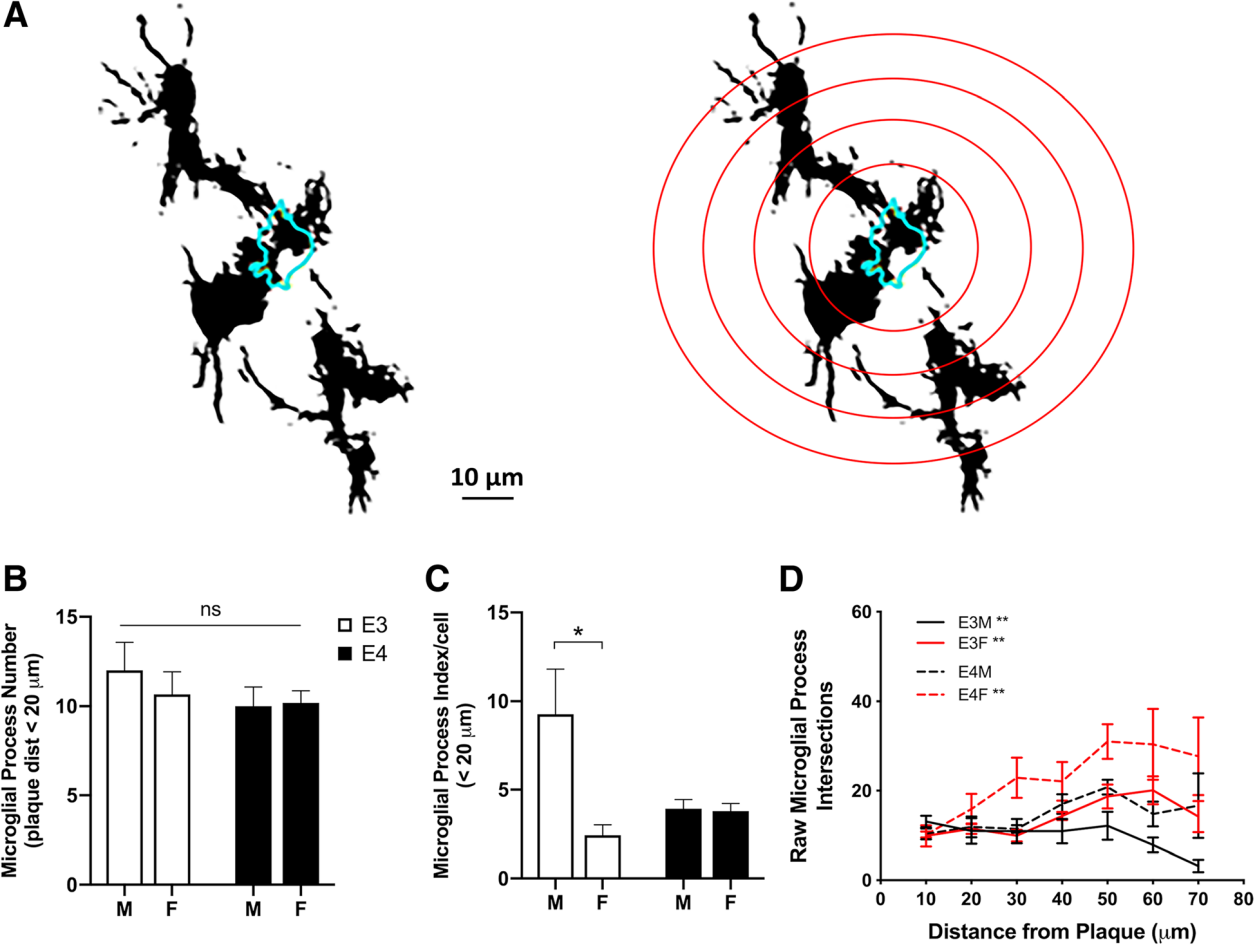
Effects of *APOE* genotype and sex on numbers of microglial processes. **a** Representative image of a thresholded confocal stack labeled with ThioS (cyan outline) and Iba1 immunoreactive microglia (black) in the HPC. The image below shows an overlay with Sholl analysis grid. **b** Microglial process number within 20 μm of ThioS-labeled plaques in male (M) and female (F) EFAD mice with *APOE3* (open bars) and *APOE4* (filled bars) genotypes. **c** Microglial process number/cell within 20 μm of ThioS-labeled plaques. **d** Quantification of microglial process intersections at increasing distances from ThioS-labeled plaques in male (black lines) and female (red lines) EFAD mice with *APOE3* (solid lines) and *APOE4* (dashed lines) genotypes. Numbers of mice analyzed per group are as follows: male E3FAD (*n* = 6), female E3FAD (*n* = 5), male E4FAD (*n* = 4), and female E4FAD (*n* = 5). ns denotes not significant, * denotes *p* < 0.05, *** denotes *p* < 0.001

Our findings demonstrate that microglial interactions with amyloid plaques differ significantly by both *APOE* genotype and sex. It is possible that the data reflect differences across *APOE* genotype and sex in the numbers of microglial processes available to interact with plaques. In this case, the pattern of increased microglia-plaque interactions observed in E3FAD males would result from a higher density of microglial processes in the plaque environment. To investigate this possibility, we used Sholl analysis to quantify the number of microglial processes adjacent to plaques in EFAD mice across *APOE* genotype and sex (Fig. 4a). Our findings show no statistically significant differences across groups in the number of microglial process segments within the immediate plaque environment (within 20 μm) (Fig. 4b). These data indicate that observed differences in microglial interactions with amyloid deposits by sex and *APOE* genotype are not explained by differences in the quantity of microglial processes in the immediate plaque environment. Interestingly, there was a significant *APOE*-sex interaction and main effect of sex (Fig. 4c, *p* = 0.04 and *p* = 0.004) on the number of microglial processes normalized to the number of Iba1-immunostained cells in the near plaque environment, suggesting increased processes on a per cell basis in E3FAD males. Further, with increasing distances away from the plaque environment, the total number microglial processes differed across groups with the lowest in E3FAD males and the highest in E4FAD females. Linear regression analyses demonstrated a significant decrease in microglia processes with increasing distance away from the plaque in E3FAD males (Fig. 4d, *p* = 0.007), but a significant and opposite relationship in E3FAD and E4FAD females (Fig. 4d, *p* = 0.003 and *p* = 0.003). Therefore, although total numbers of microglial processes in the plaque environment are similar across groups, *APOE* genotype and sex are associated with differences in the number of processes per cell in the local plaque environment and microglial process number at increasing distances away from plaques.

### *APOE* genotype and sex are associated with levels of amyloid plaques and microglial activation

**Fig. 5:**
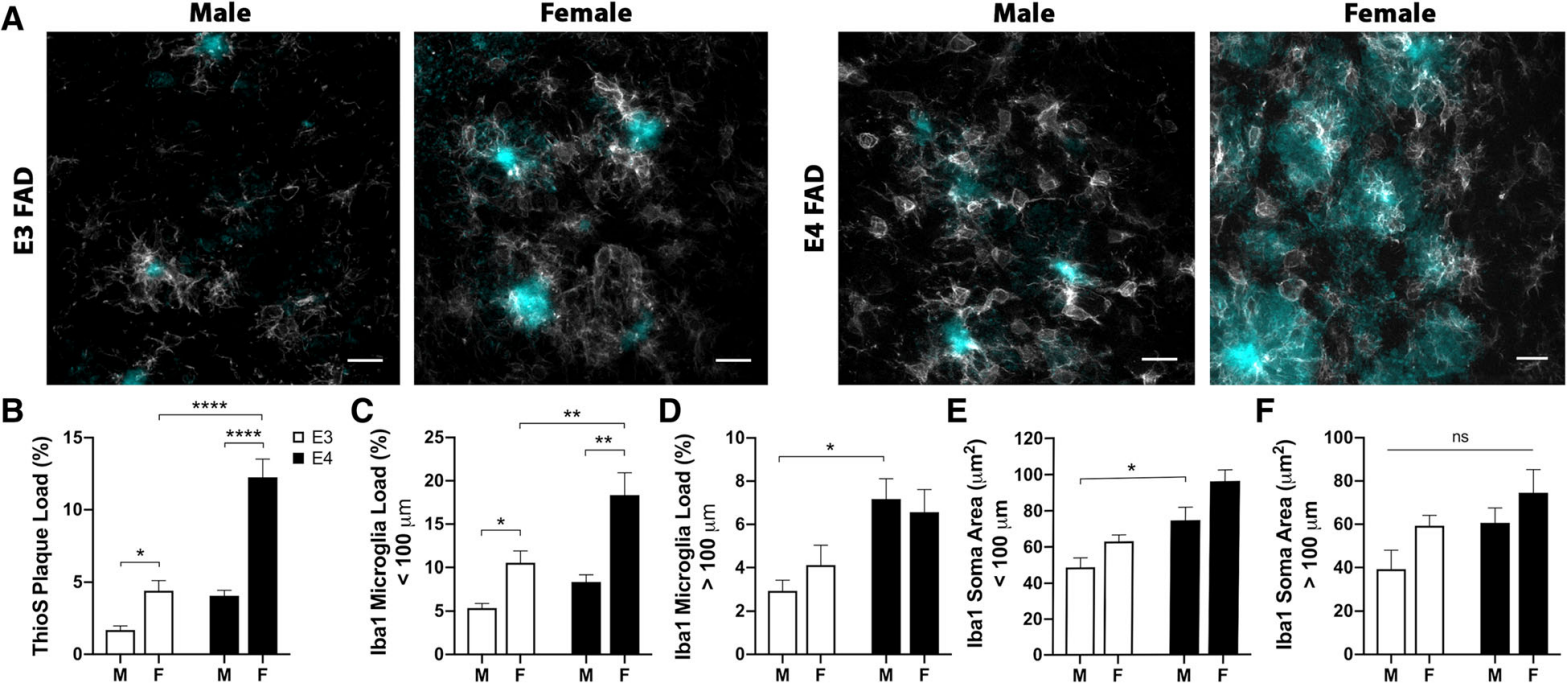
Effects of *APOE* genotype and sex on AD-related pathology. **a** Representative images show ThioS-positive plaques (cyan) and Iba-1 immunoreactive microglia (white) in the HPC of EFAD mice. Scale bars = 15 μm. **b** Quantification of amyloid plaque load (% of total ROI area) in male (M) and female (F) EFAD mice with *APOE3* (open bars) and *APOE4* (filled bars) genotypes. **c**-**d** Quantification of microglia load (% of total ROI area) near (< 100 μm, C) and far (> 100 μm, D) from ThioS-positive plaques. **e**-**f** Quantification of activated microglial phenotype (mean soma area of Iba1-immunoreactive cells) near (< 100 μm, E) and far (> 100 μm, F) from ThioS-positive plaques. Numbers of mice analyzed per group are as follows: male E3FAD (*n* = 7), female E3FAD (*n* = 7), male E4FAD (*n* = 5), and female E4FAD (*n* = 6). * denotes *p* < 0.05, ** denotes *p* < 0.01, **** denotes *p* < 0.0001, ns denotes not significant

Consistent with prior observations [7], we found that accumulation of AD-related pathology in the EFAD mouse model of AD is abundant in the subiculum and other hippocampal sub-regions and is increased by both *APOE4* genotype and female sex (Additional file 1: Figure S1). First, using ThioS, we observed that amyloid plaque burden in the HPC at 6 months of age was lowest in male E3FAD mice and highest in female E4FAD mice with comparatively moderate levels in male E4FAD and female E3FAD mice (Fig. 5a). There were significant main effects of *APOE* genotype and sex (Fig. 5b, *p* < 0.0001 and *p* < 0.0001) where *APOE4* genotype and female sex were associated with greater amyloid load. In addition, we observed a significant interaction between these two factors in which the increased amyloid burden associated with *APOE4* was significantly greater in female mice (Fig. 5b, *p* = 0.001). Similar effects were observed when analyses were restricted to the subiculum (Additional file 2: Figure S2). Next, we assessed microglial burden near (< 100 μm, Fig. 5c) and far (> 100 μm, Fig. 5d) from plaque regions. Microglial burden near plaques was significantly increased by both *APOE4* genotype and female sex (Fig. 5c, *p* = 0.001 and *p* < 0.0001, respectively) with no significant interaction between these factors. Again, similar findings were observed with data collected only from the subiculum (Additional file 2: Figure S2). In HPC ROIs greater than 100 μm away from ThioS-labeled plaques, there was a significant main effect of *APOE* genotype in male mice (Fig. 5d, *p* = 0.003), but not sex, on microglial burden. Because increased microglial soma size is a morphological indicator of activated phenotype [26], we also quantified soma size of Iba1-immunoreactive cells in ROIs both proximal (Fig. 5e) and distal to ThioS-labeled plaques (Fig. 5f). There was significant main effect of *APOE* genotype and sex (Fig. 5e, *p* < 0.0001 and *p* = 0.007, respectively) on microglia soma size, a relationship that failed to reach statistical significance for microglia more than 100 μm from amyloid plaques (Fig. 5f).

## Discussion

In this study, we examined how microglial interactions with amyloid plaques are affected by *APOE* genotype and/or sex. Several novel observations document that AD risk factors impact pathogenesis, at least in part, by regulating microglial functions. First, we observed that microglial plaque coverage in EFAD mice is significantly reduced by both *APOE4* and female sex. Consistent with prior findings that microglial plaque coverage is positively associated with plaque compaction [9], we observed that plaque circularity, an index of plaque compaction [63], is reduced by both *APOE4* and female sex relative to male E3FAD mice. Previously, microglial interactions with plaques have been shown to be TREM2 dependent [63]. In support of this we found that TREM2 expression levels in plaque-associated microglia, and specifically within their processes proximal to plaques, were lower in EFAD mice with *APOE4* or female sex relative to male E3FAD mice. Interestingly, the relationship among TREM2 expression, *APOE* genotype, and sex was not significant in microglia located away from plaques. This finding, therefore, indicates that some aspects of microglial function differ in the presence versus absence of plaques, which is consistent with recent observations of microglial heterogeneity in relation to plaque proximity [10, 41]. Importantly, numbers of microglial processes within the plaque environment were the same across *APOE* genotypes and sex, suggesting that our observed differences in microglial plaque interactions were associated not with the availability of microglial processes, but rather functional aspects of microglia in *APOE4* and female EFAD mice that may affect their ability to detect and/or interact with plaques. Consistent with this possibility, we observed that the number of processes per microglia in the near plaque environment was significantly lower in *APOE4* and female EFAD relative to male E3FAD mice. In addition, microglial burden and activation were higher in females and E4FAD mice of both sexes than in male E3FAD mice. Further, male E3FAD mice showed the lowest amyloid burden and female E4FAD the highest, a pattern opposite to that observed with microglial plaque coverage. An intriguing possibility is that increased plaque coverage may contribute to lower pathology, for example, as a result of plaque compaction and phagocytosis. Indeed, we observed a significant inverse association between plaque coverage and plaque perimeter specifically in male E3FAD mice. In support of this idea, recent findings have shown that disruption of microglial plaque coverage, resulting from AD-associated TREM2 mutations and TREM2 hemizygosity, are associated with reduced Aβ accumulation [36].

Our findings add to a growing literature indicating the importance of microglial pathways in *APOE* genotype influences on the development of AD. Across both sexes, we observed that *APOE4* was associated with increased overall Iba1 burden and activated microglial phenotype, consistent with prior observations in EFAD mice [49]. Further, we found that microglia in E4FAD mice exhibit reduced plaque coverage. To our knowledge, this is the first report to define the effects of *APOE* genotype on the recently characterized, TREM2-dependent plaque coverage by microglia. Prior studies using less specific analyses have yielded conflicting findings. Yang et al.*,* (2013) found that levels of plaque association of bone marrow-derived macrophages transplanted into irradiated APPswe/PS1Δ E9 mice were lower in macrophages from *APOE4* than from *APOE3* mice [60]. In contrast, Rodriguez et al.*,* (2014) reported greater association of microglia with cortical plaques in E4FAD versus E3FAD mice [44]. Rodriguez and colleagues also reported larger plaque size and a greater proportion of compact plaques in the subiculum of E4FAD mice in comparison to E3FAD mice [44]. Although we did not quantify plaque size and morphology in the same manner, our findings of higher levels of microglial plaque coverage and plaque circularity in male E3FAD mice may be predicted to yield smaller plaque size and a higher proportion of compact plaque morphology [63]. Apparent differences between our findings and those of Rodriguez et al.*,* may reflect important methodological variables including differences in staining (immunochemistry versus ThioS) and plaque size inclusion criteria. Perhaps most importantly, our data show that differences between E3FAD and E4FAD mice in microglial interactions with plaques are significantly affected by sex, a variable not considered in prior work.

While the mechanisms contributing to the observed regulation of microglial plaque coverage by *APOE* are unclear, interactions between *APOE* and TREM2 are increasingly recognized as significant contributors to AD-related microglial activity and represent a compelling candidate pathway for the regulation of plaque interactions [12, 59]. For example, *APOE* has been identified as a key regulator of the molecular signature of microglia via interactions with TREM2 [25, 27, 32, 35, 40]. These investigations reveal that microglia surrounding plaques differ in their morphology and molecular expression profile from microglia that are distal to plaques [25]. Importantly, an *APOE*-TREM2 pathway appears to drive the conversion of homeostatic microglia to a disease-associated phenotype [25, 27]. Because microglial actions exert both disease-promoting and protective outcomes, regulation of the *APOE*-TREM2 pathway is expected to significantly affect AD pathogenesis with the overall effect depending, in part, upon several variables including timing. A recent study found that *APOE* knockout in two different AD mouse models was associated with decreased microglial clustering around plaques as well as a reduction in plaque compaction [54], microglial actions established to be TREM2-dependent. Further, microglia-specific knockout of *APOE* in 5xFAD mice attenuated microglial transition to the disease phenotype, partially rescuing neuronal cell death [27]. How *APOE* genotype affects microglial transition to disease phenotypes via the *APOE*-TREM2 pathway is not known. Our observations of impaired microglial coverage and plaque compaction in E4FAD mice is qualitatively consistent with findings in *APOE* knockout mice [54], suggesting that apoE4 represents reduced functionality relative to apoE3 in terms of TREM2-dependent microglial actions.

We also identify novel sex differences in microglial interactions with amyloid plaques. The observed sex differences are most apparent in *APOE3* genotype in which male E3FAD mice exhibit increased microglial plaque coverage, plaque compaction, microglial TREM2 expression and reduced plaque burden in comparison to age-matched female E3FAD mice. While the specific mechanisms contributing to these observed sex differences remain to be elucidated, our findings are consistent with abundant recent evidence of wide-ranging sex differences in microglia. Indeed, microglia from male and female rodents exhibit brain-region specific differences in the numbers and activation states during development [45] that contribute to sexual differentiation of the brain [30]. Sexually dimorphic features in microglia also exist in the adult brain, including differences in the number of microglia [34] and expression of several inflammation-related factors [45]. Further, more extensive transcriptome analyses have revealed significant sex differences in adult microglia that are at least partially independent of adult sex hormone exposure [55]. As suggested by the data presented here, microglia are increasingly implicated as key regulators of sex differences in several neurological disorders including AD [15, 43]. There are numerous sex differences in AD risk, pathogenesis, and clinical manifestation that may reflect sexually dimorphic factors in both development and adulthood [39]. We speculate that these established sex differences, alongside the novel observations presented in this study, include significant contributions of, but are not limited to, microglial actions.

Collectively, these findings suggest that the AD risk factors *APOE* genotype and female sex may affect development of AD, in part, by modulating protective microglial functions. An intriguing literature defines both beneficial and deleterious actions of microglia in the context of AD [8, 17]. Classically, microglia have been viewed as contributors to AD pathogenesis, largely as a consequence of chronic neuroinflammation that is associated with states of microglial activation [17]. Both activation of microglia and indices of neuroinflammation are regulated, individually and sometimes cooperatively, by *APOE* genotype and sex [51]. In addition to driving disease progression, microglia are also able to combat AD pathogenesis, primarily via plaque interactions that can decrease plaque size and reduce levels of dystrophic neurites presumably by limiting exposure to highly toxic Aβ species [4, 9, 21, 25, 27, 52, 61, 63, 64]. The present data demonstrate that these protective microglial actions with plaques are attenuated in *APOE4* carriers and females. Thus, *APOE* genotype and sex affect both harmful and protective microglial actions. Still unclear is how the balance, or loss thereof, in the heterogeneity in microglial functions and their regulation within plaque environments varies across the disease process.

## Conclusions

In summary, our study of the EFAD mouse model of AD documents that *APOE* genotype and sex are significant regulators of microglial interactions with amyloid plaques. Although total numbers of microglial processes in the plaque vicinity are similar across sex and *APOE* genotype, protective microglial-plaque interactions, including barrier formation and plaque compaction, are maximized in a rank order opposite to pathology levels, such that male E3FAD mice exhibit the best and female E4FAD mice the poorest outcomes. These findings suggest that microglial functions, specifically microglial plaque coverage, contribute to the mechanisms by which *APOE4* genotype and female sex increase AD risk. Further investigation is required to define the mechanisms driving *APOE* and sex differences in microglial function, information that will have significant relevance to the development of therapeutic strategies that intervene in early stages of AD pathogenesis.

## Additional files

Additional file 1: **Figure S1.** Representative low magnification images demonstrate that amyloid deposition is predominantly localized to regions of hippocampus (HP) and cortex (CX) in EFAD mice. Images show ThioS-stained sagittal sections from 6 month-old male (left panels) and female (right panels) E3FAD (upper row) and E4FAD (lower row) mice. Insets show higher magnification of the subiculum region of hippocampus. (TIF 17011 kb)

Additional file 2: **Figure S2.** (A) Quantification of amyloid plaque load (% of total ROI area) in male (M) and female (F) EFAD mice in the subiculum of the HPC with *APOE3* (open bars) and *APOE4* (filled bars) genotypes. (B) Quantification of microglia load (% of total ROI area) near (< 100 μm) ThioS-positive plaques in the subiculum. (C) Quantification of TREM2 load in the subiculum near (< 100 μm) ThioS-positive plaques. * denotes *p* < 0.05, ** denotes *p* < 0.01, *** denotes *p* < 0.001, **** denotes *p* < 0.0001 (TIF 6479 kb)

## Abbreviations

AD: Alzheimer’s disease
ANOVA: Analysis of variance
APOE: Apolipoprotein E
APP: Amyloid precursor protein
Aβ: Amyloid beta
EDTA: Ethylenediaminetetraacetic acid
FAD: Familial Alzheimer’s disease
HPC: Hippocampus
Iba1: Ionized calcium binding adaptor molecule 1
PS1: Presenilin 1
ROI: Region of interest
SEM: Standard error of the mean
TBS: Tris-buffered saline
ThioS: Thioflavin S
TREM2: Triggering receptor expressed on myeloid cells 2
