# Supplementary figures and images for: APOE genotype and sex affect microglial interactions with plaques in Alzheimer’s disease mice
Source: Acta Neuropathol Commun. 2019 May 21;7:82. doi: 10.1186/s40478-019-0729-z (PMC6528326; doi:10.1186/s40478-019-0729-z)

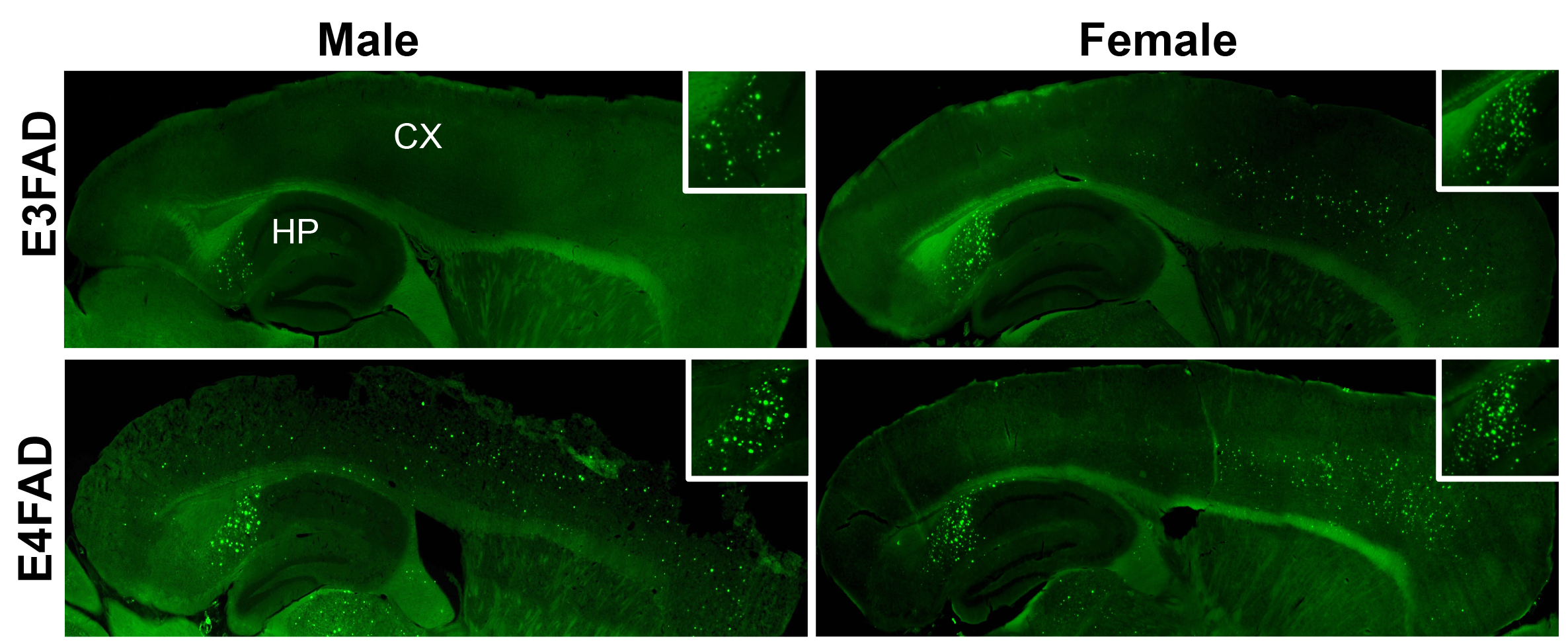

Supplement: Supplementary file 1 — Figure S1. Representative low magnification images demonstrate that amyloid deposition is predominantly localized to regions of hippocampus (HP) and cortex (CX) in EFAD mice. Images show ThioS-stained sagittal sections from 6 month-old male (left panels) and female (right panels) E3FAD (upper row) and E4FAD (lower row) mice. Insets show higher magnification of the subiculum region of hippocampus. (TIF 17011 kb) [file 40478_2019_729_MOESM1_ESM.tif]

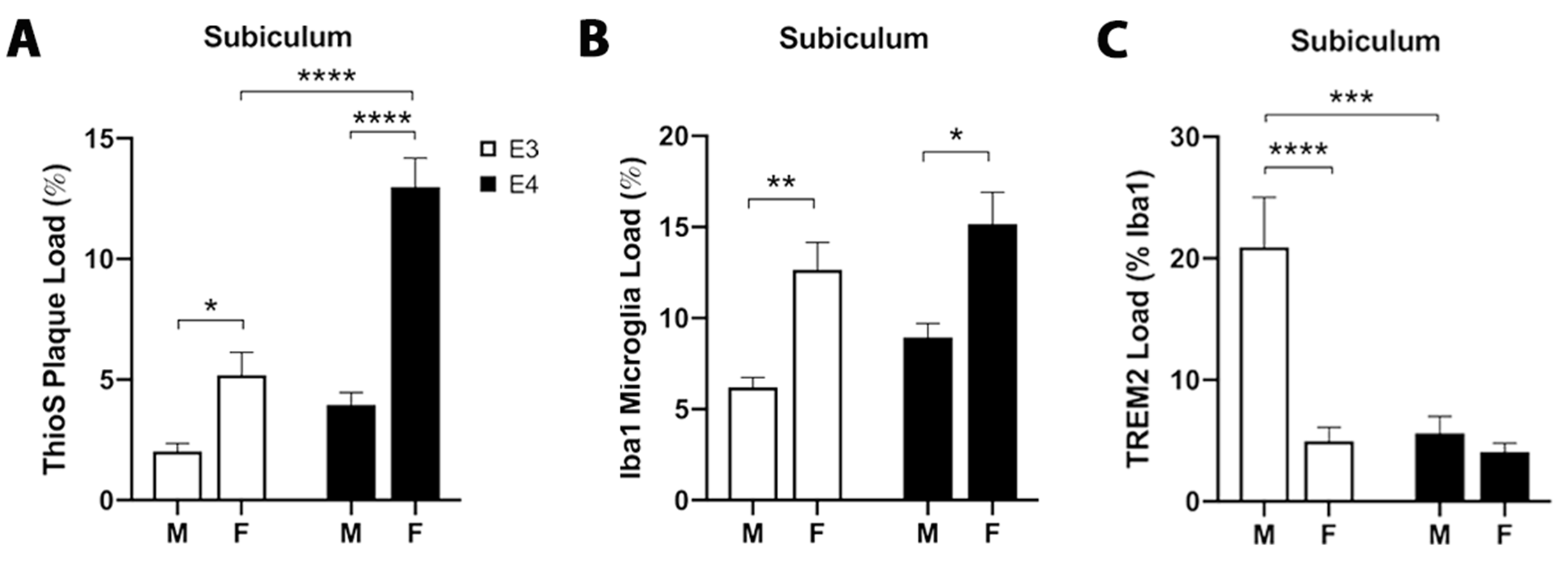

Supplement: Supplementary file 2 — Figure S2. (A) Quantification of amyloid plaque load (% of total ROI area) in male (M) and female (F) EFAD mice in the subiculum of the HPC with APOE3 (open bars) and APOE4 (filled bars) genotypes. (B) Quantification of microglia load (% of total ROI area) near (< 100 μm) ThioS-positive plaques in the subiculum. (C) Quantification of TREM2 load in the subiculum near (< 100 μm) ThioS-positive plaques. * denotes p < 0.05, ** denotes p < 0.01, *** denotes p < 0.001, **** denotes p < 0.0001 (TIF 6479 kb) [file 40478_2019_729_MOESM2_ESM.tif]
